# Identification of a novel Parkinson’s disease locus via stratified genome-wide association study

**DOI:** 10.1186/1471-2164-15-118

**Published:** 2014-02-10

**Authors:** Erin M Hill-Burns, William T Wissemann, Taye H Hamza, Stewart A Factor, Cyrus P Zabetian, Haydeh Payami

**Affiliations:** 1 Division of Genetics, Wadsworth Center, New York State Department of Health, Albany, NY, USA; 2 Department of Neurology, Emory University School of Medicine, Atlanta, GA, USA; 3 VA Puget Sound Health Care System and Department of Neurology, University of Washington, Seattle, WA, USA; 4 Department of Biomedical Science, School of Public Health, State University of New York, Albany, NY, USA

**Keywords:** GWAS, Parkinson’s disease, *SNCA*, *MAPT*, *HLA*, Genetic heterogeneity, Secondary GWAS, Stratified GWAS, Chromosome 1p

## Abstract

**Background:**

Parkinson’s disease (PD) is complex and heterogeneous. The numerous susceptibility loci that have been identified reaffirm the complexity of PD but do not fully explain it; e.g., it is not known if any given PD susceptibility gene is associated with all PD or a disease subtype. We also suspect that important disease genes may have escaped detection because of this heterogeneity. We used presence/absence of family history to subdivide the cases and performed genome-wide association studies (GWAS) in Sporadic-PD and Familial-PD separately. The aim was to uncover new genes and gain insight into the genetic architecture of PD.

**Results:**

Employing GWAS on the NeuroGenetics Research Consortium (NGRC) dataset stratified by family history (1565 Sporadic-PD, 435 Familial-PD, 1986 controls), we identified a novel locus on chromosome 1p21 in Sporadic-PD (P_NGRC_ = 4×10^-8^) and replicated the finding (P_Replication_ = 6×10^-3^; P_Pooled_ = 4×10^-10^) in 1528 Sporadic-PD and 796 controls from the National Institutes of Neurologic Disease and Stroke (NINDS) Repository. This is the fifth PD locus to be mapped to the short arm of chromosome 1. It is flanked by *S1PR1* and *OLFM3* genes, and is 200 kb from a multiple sclerosis susceptibility gene. The second aim of the study was to extend the stratified GWAS to the well-established PD genes. *SNCA_* rs356220 was associated with both Sporadic-PD (OR = 1.37, P = 1×10^-9^) and Familial-PD (OR = 1.40, P = 2×10^-5^). *HLA*_rs3129882 was more strongly associated with Sporadic-PD (OR = 1.38, P = 5×10^-10^) than Familial-PD (OR = 1.12, P = 0.15). In the *MAPT* region*,* virtually every single nucleotide polymorphism (SNP) had a stronger effect-size and lower P-value in Familial-PD (peak P = 8×10^-7^) than in Sporadic-PD (peak P = 2×10^-5^).

**Conclusions:**

We discovered and replicated a new locus for Sporadic-PD which had escaped detection in un-stratified GWAS. This demonstrates that by stratifying on a key variable the power gained due to diminished heterogeneity can sometimes outweigh the power lost to reduced sample size. We also detected distinct patterns of disease associations for previously established PD susceptibility genes, which gives an insight to the genetic architecture of the disease and could aid in the selection of appropriate study population for future studies.

## Background

PD is heterogeneous. Despite the great strides made recently, we still do not have a clear picture of the genetic architecture of PD, partly because not all the genes have been identified, and partly because we do not know if a given gene is associated with all or a subtype of PD. An important outcome of GWAS will be to use the vast information content that has been gained [1-7] to define disease subtypes based on their genetic associations. As a starting point, we posit that using surrogates for the underlying heterogeneity may help define the disease subtypes that each gene is associated with and may also reveal genes that were previously masked by this heterogeneity. Here, we use family history as a surrogate. The majority of PD (70%-85%) is non-familial (henceforth Sporadic-PD). The remaining 15%-30% of PD patients have a positive family history (Familial-PD), but rarely do their kindreds display a Mendelian inheritance pattern (Mendelian-PD). Mendelian-PD has been linked to pathogenic mutations in *SNCA*, *LRRK2*, *PARK2*, *PINK1*, *DJ1*, *ATP13A2* and *VPS35 *[8-16]. The vast majority of Familial-PD remains idiopathic. The genetic distinction between idiopathic Familial-PD and Sporadic-PD, if any exists, is unknown.

Idiopathic PD involves complex interactions between the genome and environmental exposures [6,7,17,18]. It is operationally assumed that the same set of susceptibility genes predispose to Familial and Sporadic-PD. In fact, GWAS have successfully uncovered numerous susceptibility loci without separating the subtypes [1-7]. We hypothesized that Familial-PD and Sporadic-PD have different genetic structures. We acknowledge that since genetic disease can manifest without a family history due to incomplete penetrance (e.g., *LRRK2* mutations [19]), and environmentally-induced disease can cluster in families due to common exposure, there must exist an invisible overlap between Sporadic and Familial-PD. However, they might differ in the relative burden of incompletely penetrant Mendelian genes vs. genes that confer susceptibility to environmental causes. There is evidence in the literature that supports this notion: consider three well-established PD-associated genes: *GBA*, *LRRK*2, and *HLA. GBA* mutations are significantly more common in Familial than in Sporadic-PD [20]. *LRRK2* G2019S is also significantly more common in Familial than in Sporadic-PD [21]. *HLA*, on the other hand, is more strongly associated with Sporadic-PD than with Familial-PD [3]. There are often no overt phenotypic differences between these genetic subtypes. Subtle clinical differences were only detected after the genes were identified and subtypes were defined genetically. This in itself underscores the importance of finding the genes.

Here we report the first GWAS stratified by Sporadic and Familial-PD which identified a previously unknown PD susceptibility gene in Sporadic-PD. We also present evidence for distinct patterns of associations for several well-established PD susceptibility loci with familial and sporadic subtypes.

## Methods

This study was approved by institutional review boards at the participating institutions: Albany Medical Center, Emory University, Kaiser Permanente Northwest Division, New York State Department of Health, Oregon Health & Sciences University (OHSU) and the Department of Veterans Affairs VA Puget Sound Health Care System (VAPSHCS). All study participants gave informed consent. All patients and most control subjects gave written signed consent; a portion of control subjects, recruited at OHSU and VAPSHCS, who wished to remain anonymous read the written informed consent and gave verbal consent as approved by the institutional review boards at OHSU and VAPSHCS respectively. All participants were adults and gave consent on their own behalf. No parents or guardians were asked to consent for the subjects. Subjects were from NGRC and included 2000 persons with PD (435 Familial-PD, 1565 Sporadic-PD) and 1986 controls (Additional file 1). PD was diagnosed by movement disorder specialists using UK/NINDS diagnostic criteria [22]. Controls were free of neurodegenerative disease, 340 of them were examined by a neurologist. All 3986 subjects were confirmed as genetically unrelated (PI_HAT ≤ 0.15). Familial-PD was designated for cases with one or more first or second-degree relatives with PD; Sporadic-PD was all other cases. There was no significant difference between Familial and Sporadic cases in age at recruitment, age at onset, gender, percentage of Ashkenazi Jews, and the inverse association of smoking and coffee with PD (Additional file 1).

The genome-wide genotype data were generated by our group and are publically available on dbGaP (http://www.ncbi.nlm.nih.gov/gap). Standardized subject selection criteria, protocols and subject characteristics were used which are published [3] and are also available online (http://www.ncbi.nlm.nih.gov/gap) with complete genotype and phenotype data on the entire NGRC cohort (phs000196.v2.p1). Genome-wide genotyping was performed on DNA from whole blood using the Illumina HumanOmni1-Quad_v1-0_B array [3]. 811,597 SNPs passed quality-control (minor-allele frequency ≥0.01, call-rate ≥99%, Hardy-Weinberg P ≥ 10^-6^, allele-frequency difference in men vs. women ≤0.15, missing rate in cases vs. controls P ≥ 10^-5^). An additional 6.4 million SNPs with frequency ≥0.01 were imputed with high fidelity (info-score ≥90%) using the 1000 Genomes Phase I integrated variant set release v3 and the IMPUTE v.2.2.2 software [23]. The analyses were conducted on the total of 7.2 million SNPs. For rs2338971 (the top signal on chromosome 1), the call rate for individuals with imputed genotype probability ≥90% was 0.98 in cases and 0.98 in controls. Genotype frequencies in controls for rs2338971 were in Hardy Weinberg equilibrium (P = 0.24).

For replication, we used cases and controls from the NINDS Human Genetics DNA and Cell Line Repository (http://ccr.coriell.org/ninds). We obtained the NINDS GWAS data [2] from dbGaP (http://www.ncbi.nlm.nih.gov/projects/gap/cgi-bin/study.cgi?study_id=phs000089.v3.p2). We used only white non-Hispanic subjects. The sample size was 924 cases (621 Sporadic-PD, 303 Familial-PD) and 797 controls. We imputed the two SNPs of interest on chromosomes 1 and 8 with high confidence (info-score ≥ 0.97). In addition, we had purchased 1490 PD DNA samples (1025 Sporadic, 465 Familial) from the NINDS Repository. We directly genotyped the DNA samples using TaqMan. The GWAS dataset and our DNA samples had only 120 cases in common. This allowed us to combine the non-overlapping segments of the two datasets to attain a larger sample size, as well as an opportunity to validate imputation vs. genotyping results using the overlapping 120 cases. The genotypes, TaqMan vs. imputation, for the top signal at chromosome 1 matched 100% for these 120 individuals. Call rates were 0.95 for genotyped cases, 0.97 for imputed cases with genotype probability ≥90%, and 0.98 for imputed controls with genotype probability ≥90%. Controls were in Hardy-Weinberg proportions (P = 0.11). The final sample size for replication was 2235 cases (1528 Sporadic, 707 Familial) and 796 controls, all from the NINDS Repository.

Statistical analyses for GWAS were performed using ProbABEL v.0.1-9d [24] adjusting for age at blood draw, sex and two principal components; and using R version 3.0.1 (http://www.r-project.org/) for replication adjusting for sex and study. Differences in allele frequencies of Sporadic vs. Familial cases were tested using logistic regression. Conditional analysis was conducted using logistic regression. P values shown for replication of chromosome 1 signal are one-sided, due to the directionality of the hypotheses being tested [25]. Linkage disequilibrium (LD) was assessed using Haploview V-4.2 [26] and LocusZoom [27].

We checked the 44 SNPs in the chromosome 1 peak (those with P < 10^-5^) for evidence of association with gene expression in expression quantitative trait loci (eQTL) databases (http://www.sanger.ac.uk/resources/software/genevar/, http://eqtl.uchicago.edu/cgi-bin/gbrowse/eqtl/, http://gbrowse.csbio.unc.edu/cgi-bin/gb2/gbrowse/seeqtl/, http://www.ncbi.nlm.nih.gov/gtex/GTEX2/gtex.cgi, http://www.scandb.org/newinterface/about.html) and in published eQTL studies for variants associating with gene expression in the brain [28-32].

## Results

### Known loci

**Figure 1:**
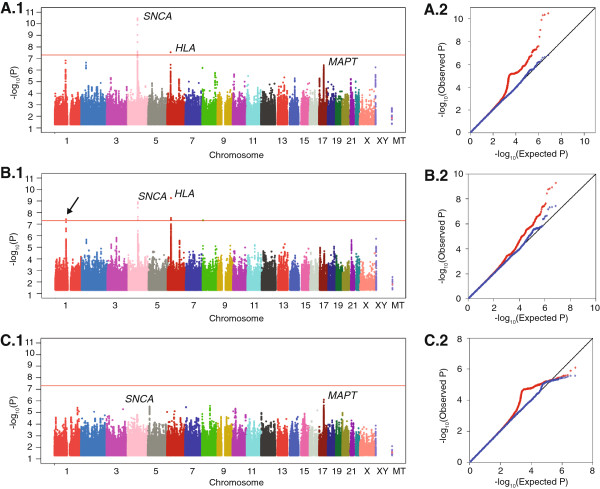
**Genome-wide association P values for All-PD (A), Sporadic-PD (B) and Familial-PD (C).** The Manhattan plots (A.1, B.1, C.1) show the -Log_10_ P values for association of SNPs with PD within each stratification, plotted according to their location across each chromosome. The horizontal red line is P = 5×10^-8^. The quantile-quantile (QQ) plots (A.2, B.2, C.2) depict the distribution of expected P-values for no disease association (black line) vs. the observed P-values for all SNPs genome-wide (red line), and excluding *SNCA* (Chr 4 - bp: 90453000 to 91867000), *HLA* (Chr 6 - bp: 30615000 to 32963000) and *MAPT* (Chr 17 - bp: 42285000 to 44866000) (blue line).

When GWAS data were analyzed without stratification (All-PD with 2000 cases, 1986 controls), we recovered *SNCA* as the strongest signal; *HLA*, which also reached genome-wide significance; and *MAPT,* which had a clear peak but fell below the significance threshold (Figure 1A, Table 1). It is well established that polymorphisms in *SNCA, MAPT,* and *HLA* are associated with PD; it is not known however if their effects are ubiquitous across all PD or stronger in Sporadic or Familial subtype.

**Table 1:** Results of GWAS conducted in All-PD and stratified by Sporadic-PD and Familial-PD

**CHR *Gene***	**SNP**	Base pair position*	**Minor/major allele**	**Allele frequency**				**Association with PD**								
				**Case**			**Control**	**All-PD**			**Sporadic-PD**			**Familial-PD**		
				**All-PD**	**Sporadic**	**Familial**		**OR**	**SE**	**P**	**OR**	**SE**	**P**	**OR**	**SE**	**P**
**Known highly significant loci**																
4 *SNCA*	rs356220	90641340	T/C	0.440	0.439	0.443	0.364	1.38	0.07	3×10^-11^	1.37	0.07	1×10^-9^	1.40	0.11	2×10^-5^
6 *HLA*	rs3129882	32409530	G/A	0.459	0.470	0.418	0.395	1.31	0.06	3×10^-8^	1.38	0.07	5×10^-10^	1.12	0.09	0.15
17 *MAPT*	rs199498	44865603	C/T	0.190	0.197	0.162	0.236	0.74	0.05	2×10^-6^	0.78	0.05	2×10^-4^	0.59	0.06	8×10^-7^
**New Signals in NGRC**																
1	rs2338971	101880005	T/C	0.187	0.179	0.213	0.232	0.74	0.04	2×10^-7^	0.71	0.04	4×10^-8^	0.86	0.08	0.12
8	rs12681349	4277990	T/C	0.376	0.369	0.403	0.426	0.78	0.04	7×10^-7^	0.75	0.04	5×10^-8^	0.88	0.07	0.10
**Replication**																
1	rs2338971	101880005	T/C	0.184	0.182	0.188	0.212	0.82	0.06	5×10^-3^	0.81	0.07	6×10^-3^	0.83	0.08	0.03
8	rs12681349	4277990	T/C	0.427	0.420	0.442	0.419	1.02	0.06	0.38	0.99	0.06	0.41	1.09	0.08	0.13
**Pooled NGRC and Replication**																
1	rs2338971	101880005	T/C	0.185	0.181	0.197	0.227	0.76	0.03	5×10^-10^	0.74	0.04	4×10^-10^	0.82	0.05	2×10^-3^

*Genome Build 37.

The top hit for *SNCA* region was rs356220 at the 3′ of the gene (All-PD: OR = 1.38, P = 3×10^-11^). This variant was also the most significant marker in both Sporadic-PD (OR = 1.37, P = 1×10^-9^) and Familial-PD (OR = 1.40, P = 2×10^-5^). There was no difference in the *SNCA* rs356220 allele frequencies in Familial and Sporadic-PD (P = 0.85).

The top hit for *HLA* was rs3129882 in intron 1 of *HLA-DRA* (All-PD: OR = 1.31, P = 3×10^-8^). This association was strong in Sporadic-PD (OR = 1.38, P = 5×10^-10^) but weak and statistically non-significant in Familial-PD (OR = 1.12, P = 0.15). The difference between Sporadic and Familial-PD in the frequency of *HLA-DRA* rs3129882 alleles was significant (P = 6×10^-3^).

Within the *MAPT* region, defined from 43.5 Mb to 44.9 Mb on chromosome 17 (Human Genome Build 37) and including *PLEKHM1*, *MAPT*, *NSF* and *WNT3* genes, 2,365 SNPs gave evidence for association with PD (P < 10^-5^ in All-PD). The effect sizes for the 2,365 SNPs were always greater in Familial-PD (0.56 ≤ OR ≤ 0.68) than for Sporadic-PD (0.76 ≤ OR ≤ 0.81). Moreover, nearly every SNP (2,363 of 2,365) achieved higher statistical significance in Familial-PD (2×10^-4^ ≥ P ≥ 8×10^-7^) than in Sporadic-PD (8×10^-4^ ≥ P ≥ 2×10^-5^). The GWAS signal for the *MAPT* region peaked at P = 8×10^-7^ in Familial-PD vs. P = 2×10^-5^ in Sporadic-PD. The sample size for Sporadic-PD was 3-times larger than Familial-PD and therefore power was not a limiting factor in this case because the association was strong in Familial-PD despite the smaller sample size.

### New locus

The GWAS on Sporadic-PD (1565 cases vs. 1986 controls) revealed two signals, (Figure 1B, Table 1), one on the short arm of chromosome 1 (a SNP-dense peak with top signal at rs2338971, P = 4×10^-8^) and another on chromosome 8 (a single SNP, rs12681349, P = 5×10^-8^). The GWAS on Familial-PD (435 cases vs. 1986 controls) did not reveal any statistically significant signals (Figure 1C).

We tested the new Sporadic-PD signals in an additional 1528 Sporadic-PD cases, 707 Familial-PD cases and 796 controls from the NINDS Repository (Table 1). The signal on chromosome 1 replicated. In Sporadic-PD, the top chromosome 1 SNP, rs2338971, gave P_Replication_ = 6×10^-3^ with OR = 0.81 which was in the same direction as NGRC and therefore boosted the significance of the combined data to P_Pooled_ = 4×10^-10^. The chromosome 8 signal did not replicate (Table 1).

The confirmed signal maps to the short arm of chromosome 1 at p21. The signal is a strong peak with 44 SNPs that achieved 4×10^-8^ ≤ P < 10^-5^ for association with Sporadic-PD in the NGRC dataset. We defined the new locus based on the location of these 44 SNPs as a 141 kilo base-pair (kb) region from base-pair (bp) 101,872,292 to 102,013,715.

We performed sensitivity analysis testing association of the top SNP, rs2338971, within subgroups of Sporadic-PD classified by sex, age at onset, genotype (*SNCA, HLA* and *MAPT*), exposure (smoking, caffeinated-coffee, non-steroidal anti-inflammatory drugs use), recruitment site, European countries of ancestral origin, and Ashkenazi Jewish heritage. Association of rs2338971 with Sporadic-PD was robust in every stratum with no evidence of heterogeneity across strata (Additional file 2).

**Figure 2:**
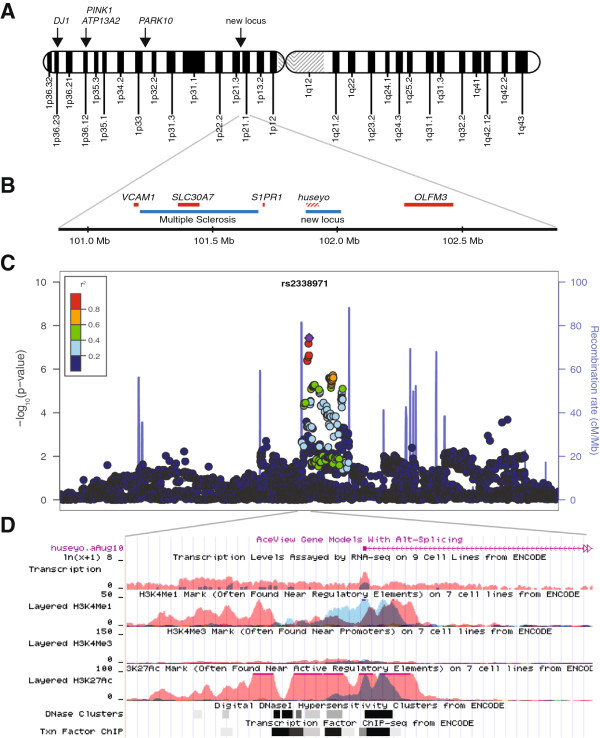
**New region of association with Sporadic-PD. (A)** Locations of PD-associated loci on the short arm of chromosome 1. **(B)** Enlargement of chromosomal region centered on the new signal and extending 1 Mb in each direction. Red bars are known genes. Hashed red bar, *huseyo*, is poorly annotated. Not all known/putative genes in the region are displayed. Blue bars are disease loci that have been mapped but the genes have not been characterized yet. **(C)** The LD structure of Sporadic-PD associated SNPs in the 2 Mb region. SNPs within 1 Mb on either side of top SNP, rs2338971, are plotted, showing –Log_10_ P values for their association with Sporadic-PD. **(D)** ENCODE data from UCSC Genome Browser showing evidence of regulatory sequences.

Four PD loci have been mapped to chromosome 1p, namely, *PARK6/PINK1*, *PARK7/DJ1*, *PARK9/ATP13A2*, and *PARK10* (Figure 2A). The new locus is more than 45 mega base-pairs (Mb) away from the closest known PD locus, which is *PARK10*. We tested LD between the SNPs that reached P < 10^-5^ for association with Sporadic-PD (44 SNPs) and SNPs that were in or within ±10 kb of *DJ1* (66 SNPs), *PINK1* (158 SNPs), *ATP13A2* (107 SNPs) and *PARK10* (15,796 SNPs). There was no correlation between the new locus and the known loci (r^2^ = 0). We therefore conclude that the signal identified here represents a previously unknown PD susceptibility locus.

The associated region maps onto a poorly-annotated gene, *huseyo* (Figure 2B). *Huseyo* is transcribed [33,34] but little is known about the putative protein. rs10493953, which gave OR = 0.64 with P = 1×10^-5^ for association with Sporadic-PD is predicted to cause a non-synonymous substitution (A/G → Q13R) in the putative protein. However, when we conditioned on the strongest signal (rs2338971), the signal for the amino-acid changing SNP was lost (P = 10^-5^ dropped to P = 0.07). The top SNP, rs2338971, also dropped in significance but was not abolished when conditioned on the amino-acid changing SNP (P = 4×10^-8^ to P = 2×10^-4^). These results suggest neither of these SNPs can explain the association signal entirely, rather they are probably tagging an as yet unknown disease-associated factor in the region.

There are several genes in the region (Figure 2B) including *VCAM1*, *SLC30A7, S1PR1* and *OLFM3* that could be considered plausible candidate genes for PD based on their known functions (see Discussion). To explore the possibility that the new signal is tagging a linked gene, we examined the pattern of LD in the region (Figure 2C). Using rs2338971 as the anchor we found that strong LD (r^2^ ≥ 0.8) extended only 8 kb telomeric and 16 kb centromeric; moderate LD (r^2^ ≥ 0.5) extended 8 kb and 100 kb and marginal LD (r^2^ ≥ 0.2) extended 30 kb and 157 kb. Therefore, even the closest genes to this signal (*S1PR1* at ~165 kb telomeric and *OLFM3* at *~*250 kb centromeric) were outside the region of marginal LD with it. To be specific, there was no LD between the 44 SNPs of the new signal and SNPs in or within ±10 kb of *OLFM3* (86 SNPs tested, r^2^ = 0) or *S1PR1* (11 SNPs tested, 0 ≤ r^2^ ≤0.08).

The block of SNPs that showed the strongest association with Sporadic-PD (5×10^-8^ < P < 5×10^-7^) overlaps with a DNA sequence that contains strongly marked regulatory regions in the ENCODE database [34] (Figure 2D). We searched five eQTL databases and published expression data on human brain (see Methods) and did not find evidence for any of the SNPs being an eQTL.

## Discussion

Although PD is recognized as a complex and heterogeneous disease, it has been treated as a single entity in most prior GWAS. While GWAS have been enormously successful, the discoveries to date are only a fraction of the information content that these large and expensive datasets encompass. In this paper, we show that accounting for aspects of disease heterogeneity, in this case family history, could give new insights into the genetic architecture of disease. Stratifying by family history, we discovered and replicated a new locus for Sporadic-PD which had escaped detection in un-stratified GWAS. We also detected distinct patterns of disease associations for some of the previously established PD susceptibility genes. We did not detect any new signals for Familial-PD, which is not surprising because the sample size was small. In order for this approach to work, the power gained by reducing heterogeneity must outweigh the power lost to decreased sample size.

This is the fifth PD locus to be mapped to chromosome 1p, which including *DJ1*, *ATP13A2*, *PINK1*, and *PARK10*, was already coined as a PD hot spot. A multiple sclerosis (MS) susceptibility locus [35] also maps to chromosome 1p, only ~200 kb away from the signal for PD. The gene for MS has not been identified. There are several plausible candidate genes for PD in the region (Figure 2). *S1PR1* (sphingosine-1-phosphate receptor 1) encodes a lipid G protein-coupled receptor involved in cell-cell adhesion, which affects differentiation of endothelial cells and has a crucial role in immune response [36,37]. *VCAM1* (vascular cell adhesion molecule 1) mediates leukocyte-endothelial cell adhesion and signal transduction, and is critical for adult neurogenesis by maintaining the structure and function of the adult forebrain subventricular zone where neuronal stem cells give rise to neurons [38]. *SLC30A7* (solute carrier family 30 member 7) encodes the zinc transporter 7 protein (ZNT7) [39]. Zinc is required for synaptic neurotransmission and can also act as an antioxidant [40]. Zinc accumulates selectively in the substantia nigra of PD brains [41,42], and in rodents, is shown to both enhance and reduce excitability of dopaminergic neurons [43]. ENCODE data [34] show strong markings within the most significant region of PD association that are predicted to be transcription factor binding sites, DNase hypersensitivity clusters, and H3K27ac histone marks, which indicate active enhancers of gene expression [44]. We did not find compelling evidence for the PD-associated SNPs being eQTL. The expression data and *in-silico* methods for combining disease-association and expression data are evolving rapidly. Studies agree that eQTL patterns vary across tissues and by cell type [30,45,46]. Thus future studies will require not only more enriched eQTL datasets (only a fraction of regulatory elements has been identified) but also a larger variety of tissues and specific cell types.

Prior GWAS have identified over 20 susceptibility loci for PD (Additional file 3), of which *SNCA, MAPT* and *HLA* have the strongest signals in our data. There is compelling evidence to suggest that association of PD with *SNCA* and *HLA* involves variations in gene expression [47-49]. In contrast, there is little information on the underlying mechanism of the association of PD with *MAPT*. Here, we detected distinct patterns in Sporadic and Familial subtypes. *SNCA* was associated with All-PD*, MAPT* was associated primarily with Familial-PD*,* and *HLA* was associated only with Sporadic-PD. We also explored differential associations with Familial vs. Sporadic-PD for all genome-wide significant signals from published studies (Additional file 3). These distinct patterns can help to generate new hypotheses and to select subtypes of PD for specific research questions. The result for *SNCA* would suggest an important and central role for this gene in all forms of PD, which is not a novel thought, considering that α-synuclein is a major component of Lewy-bodies and a ubiquitous diagnostic hallmark of PD. We hypothesize that the stronger association of *MAPT* with Familial-PD is indicative of an incompletely penetrant genetic factor, and that pedigrees may be more informative in studies of *MAPT* and PD. Whereas the seemingly exclusive association of *HLA* with Sporadic-PD suggests that that risk allele is not a cause of disease, rather it might be a genetic susceptibility to an environmental agent. Thus, we suggest that consideration of PD-relevant *HLA* markers may be particularly relevant to studies of epidemiology, exposures and infectious origins of PD.

## Conclusions

We discovered and replicated a new locus for Sporadic-PD which had escaped detection in un-stratified GWAS. This demonstrates that by stratifying on a key variable the power gained due to diminished heterogeneity can sometimes outweigh the power lost to reduced sample size. We also detected distinct patterns of disease associations for previously established PD susceptibility genes, which gives an insight to the genetic architecture of the disease and could aid in the selection of appropriate study population for future studies.

### Availability of supporting data

The data sets supporting the results for the discovery phase (NGRC dataset) were generated by our group and are publically available in the NCBI dbGaP repository, phs000196.v2.p1, (http://www.ncbi.nlm.nih.gov/projects/gap/cgi-bin/study.cgi?study_id=phs000196.v2.p1). The data sets supporting the results for the replication phase (NINDS dataset) were in part generated by us by direct genotyping of DNA samples from PD cases which we purchased from the NINDS Human Genetics Resource Center DNA and Cell Line Repository (http://ccr.coriell.org/ninds) and in part generated by Simon-Sanchez et al. [2] and are publically available in the NCBI dbGaP repository, phs000089.v3.p2 (http://www.ncbi.nlm.nih.gov/projects/gap/cgi-bin/study.cgi?study_id=phs000089.v3.p2).

## Abbreviations

PD: Parkinson’s disease; GWAS: Genome-wide association study; NGRC: NeuroGenetics Research Consortium; NINDS: National Institutes of Neurologic Disease and Stroke; SNP: Single nucleotide polymorphism; kb: Kilo-base pairs; bp: Base pair; Mb: Mega-base pairs; LD: Linkage disequilibrium; eQTL: Expression quantitative trait locus.

## Competing interests

The authors declare that they have no competing interests.

## Authors’ contributions

EMH performed data analysis and helped write the manuscript. WTW assisted with data analysis. THH assisted with data analysis. SAF helped recruit subjects for the study. CPZ helped recruit subjects for the study. HP designed the study, coordinated data collection, supervised data analysis and wrote the manuscript. All authors read and approved the final manuscript.

## Funding

We would like to acknowledge the persons with PD, their families and healthy volunteers who participated in this study. The project was supported by Award Number R01NS36960 from the National Institute of Neurological Disorders And Stroke. Additional support was provided by a Global Genetic Consortium Grant from the Michael J Fox Foundation for Parkinson’s Disease Research, Merit Review Award from the Department of Veterans Affairs (1I01BX000531), National Institutes of Aging (P30AG08017), Office of Research & Development, Clinical Sciences Research & Development Service, Department of Veteran Affairs, The Intramural Research Program of the NIH at National Library of Medicine, and the Close to the Cure Foundation. Genotyping services were provided by the Center for Inherited Disease Research (CIDR), which is fully funded through a federal contract from the National Institutes of Health to The Johns Hopkins University, contract number HHSN268200782096C. This study used samples from the NINDS Human Genetics Resource Center DNA and Cell Line Repository (http://ccr.coriell.org/ninds), as well as clinical data. Funding for NINDS-Genome-Wide Genotyping in Parkinson's Disease which generated the GWAS used for replication was provided by NINDS, and the GWAS data were obtained from the NINDS database at (http://www.ncbi.nlm.nih.gov/gap) accession number phs000089.v3.p2. The content is solely the responsibility of the authors and does not necessarily represent the official views of the funding agencies.

## Supplementary Material

Additional file 1: Table S1 NGRC Subject characteristics. Click here for file

Additional file 2: Table S2 Association of rs2338971 (chromosome 1p21) with Sporadic-PD was consistent across disease- and study-related strata. Click here for file

Additional file 3: Table S3 Previously reported genome-wide significant signals stratified by family history. Click here for file
