# Supplementary material for: Identification of a novel Parkinson’s disease locus via stratified genome-wide association study
Source: BMC Genomics. 2014 Feb 10;15:118. doi: 10.1186/1471-2164-15-118 (PMC3925254; doi:10.1186/1471-2164-15-118)
Supplement: Additional file 1: Table S1 — NGRC Subject characteristics. [file 1471-2164-15-118-S1.pdf]

Supplementary Table 1. NGRC Subject characteristics.

	Sporadic-PD	Familial-PD	Controls
N subjects	1565	435	1986
Age at onset (mean ± s.d.)	58.83 ± 11.79	56.56 ± 12.30	-
Age at blood draw (mean ± s.d.)	67.59 ± 10.68	66.06 ± 10.57	70.32 ± 14.09
Male : Female	1063 : 502	283 : 152	769 : 1217
Cigarette smoking (odds ratio ± s.e.)	0.84 ± 0.07	0.74 ± 0.09	Ref
Caffeinated coffee (odds ratio ± s.e.)	0.66 ± 0.06	0.65 ± 0.09	Ref
Ashkenazi Jewish	4.22%	5.29%	2.01%

Smoking: ever vs. never smoked >100 cigarettes in lifetime.

Coffee: Heavy vs. light. Number of cups of caffeinated coffee drank per day multiplied by the number of years of consumption; heavy and light divided at the median in controls.

Jewish/Non-Jewish: Defined by self report superimposed on principal component analysis. The core of the Jewish cluster was defined within $0.04 \leq PC1 \leq 0.055$ and $0.001 \leq PC2 \leq 0.013$.

Odds ratios (OR) were calculated for sporadic PD vs. all controls and Familial PD vs. all controls.

Smoking and coffee odds ratios include age at blood draw and sex as covariates.
