# Supplementary material for: Identification of a novel Parkinson’s disease locus via stratified genome-wide association study
Source: BMC Genomics. 2014 Feb 10;15:118. doi: 10.1186/1471-2164-15-118 (PMC3925254; doi:10.1186/1471-2164-15-118)
Supplement: Additional file 2: Table S2 — Association of rs2338971 (chromosome 1p21) with Sporadic-PD was consistent across disease- and study-related strata. [file 1471-2164-15-118-S2.pdf]

Supplementary Table 2. Association of rs2338971 (chromosome 1p21) with Sporadic-PD was consistent across disease- and study-related strata.

	N		MAF					
	**case**	**control**	**case**	**control**	**OR**	**SE (OR)**	**P**	**P_{Het}**
All subjects	1565	1986	0.18	0.23	0.71	0.04	$3.72x10^{-8}$	
**PD-associated risk factors**								
Male	1063	769	0.19	0.24	0.74	0.06	$2.53x10^{-4}$	0.36
Female	502	1217	0.16	0.23	0.66	0.07	$2.44x10^{-5}$	
Early onset (≤ 50 yrs)	381	1986	0.18	0.23	0.65	0.07	$1.62x10^{-4}$	NC
Late onset (> 50 yrs)	1183	1986	0.18	0.23	0.72	0.05	$1.70x10^{-6}$	
Smokers	581	696	0.17	0.24	0.68	0.07	$1.98x10^{-4}$	0.66
Non-smokers	673	810	0.18	0.23	0.72	0.07	$1.03x10^{-3}$	
Coffee-heavy	405	387	0.18	0.24	0.69	0.09	$4.03x10^{-3}$	0.74
Coffee-light	739	544	0.17	0.23	0.65	0.07	$4.12x10^{-5}$	
OTC NSAIDs - ever	758	657	0.16	0.23	0.64	0.06	$7.22x10^{-6}$	0.58
OTC NSAIDs - never	479	326	0.20	0.26	0.69	0.09	$3.37x10^{-3}$	
**Recruitment site**								
New York	351	300	0.17	0.22	0.71	0.10	0.02	0.99
Oregon	342	898	0.19	0.23	0.74	0.09	0.01	
Georgia	182	113	0.17	0.22	0.74	0.15	0.15	
Washington	690	675	0.18	0.24	0.67	0.07	$9.16x10^{-5}$	
**PD-associated genes**								
rs356220 (*SNCA*) - CC	493	813	0.19	0.24	0.73	0.08	$2.84x10^{-3}$	0.93
rs356220 (*SNCA*) - CT	770	901	0.17	0.23	0.68	0.06	$3.10x10^{-5}$	
rs356220 (*SNCA*) - TT	302	272	0.19	0.24	0.74	0.12	0.06	
rs3129882 (*HLA*) - AA	434	726	0.17	0.23	0.67	0.08	$4.90x10^{-4}$	0.92
rs3129882 (*HLA*) - AG	790	948	0.19	0.24	0.73	0.06	$3.04x10^{-4}$	
rs3129882 (*HLA*) - GG	341	310	0.17	0.22	0.70	0.10	0.02	
rs1378358 (*MAPT*) - CC	1059	1198	0.18	0.24	0.67	0.05	$4.58x10^{-7}$	0.82
rs1378358 (*MAPT*) - CT	434	692	0.18	0.22	0.75	0.09	0.01	
rs1378358 (*MAPT*) - TT	44	76	0.17	0.24	0.60	0.22	0.17	
**Ashkenazi Jewish**								
Yes	66	40	0.14	0.19	0.85	0.34	0.68	0.64
No	1499	1946	0.18	0.23	0.71	0.05	$5.02x10^{-8}$	
**Paternal or Maternal Ancestry**								
Great Britain	416	356	0.18	0.21	0.72	0.10	0.02	0.90
Germany / Austria	351	269	0.20	0.23	0.73	0.11	0.04	
Ireland	197	162	0.15	0.23	0.64	0.14	0.04	
Scandinavia	175	146	0.16	0.24	0.54	0.12	$4.69x10^{-3}$	
Eastern Europe	72	78	0.22	0.25	0.94	0.26	0.83	
Italy	71	68	0.13	0.24	0.45	0.17	0.04	
France	65	68	0.18	0.24	0.57	0.18	0.08	
Russia	49	23	0.17	0.30	0.54	0.25	0.19	

Analyses were adjusted for PC1, PC2, sex and age. OR=odds ratio. SE=standard error of OR. P=statistical significance of association. P_{Het}=statistical significance of heterogeneity within each strata (NC=not calculated because the two strata shared the same controls). **Smoker**: >100 cigarettes in lifetime. **Coffee**: Number of cups of caffeinated coffee drank per day multiplied by the number of years of consumption; high and low divided at the median in controls. **OTC NSAIDs**: Ever or never use of over the counter NSAIDs. **Jewish/Non-Jewish**: Defined by self report superimposed on principal component analysis. The core of the Jewish cluster was defined within 0.04≤PC1≤0.055 and 0.001≤PC2≤0.013. **Recruitment site:** US states where subjects were recruited from. **Paternal or maternal ancestry**: Self reports of the countries from which ancestors immigrated to US.
