# Supplementary material for: Identification of a novel Parkinson’s disease locus via stratified genome-wide association study
Source: BMC Genomics. 2014 Feb 10;15:118. doi: 10.1186/1471-2164-15-118 (PMC3925254; doi:10.1186/1471-2164-15-118)
Supplement: Additional file 3: Table S3 — Previously reported genome-wide significant signals stratified by family history. [file 1471-2164-15-118-S3.pdf]

Supplementary Table 3. Previously reported genome-wide significant signals stratified by family history

				Original report			NGRC All-PD			Familial-PD			Sporadic-PD		
Chr	Gene	SNP [Ref]	BP	OR	SE	P	OR	SE	P	OR	SE	P	OR	SE	P
1	*GBA*	rs2230288 [1]	155206167	0.02	1.71	5E-8	1.57	0.27	9E-3	1.94	0.49	8E-3	1.49	0.27	0.03
1	*PARK16*	rs708723 [2]	205739266	0.44	0.91	1E-12	0.88	0.04	6E-3	0.85	0.07	0.04	0.89	0.05	0.02
2	*STK39*	rs2102808 [3]	169117025	0.13	1.28	2E-11	1.20	0.08	8E-3	1.31	0.14	0.01	1.17	0.09	0.03
3	*NMD3*	rs34016896 [2]	160992864	0.31	1.14	2E-8	1.10	0.06	0.05	0.98	0.08	0.83	1.15	0.06	0.01
3	*MCCC1/LAMP3*	rs10513789 [4]	182760073	0.20	0.80	3E-10	0.95	0.06	0.40	1.00	0.10	1.00	0.93	0.06	0.25
3	*MCCC1/LAMP3*	rs11711441 [3]	182821275	0.14	0.82	1E-8	0.91	0.06	0.17	0.90	0.11	0.39	0.90	0.07	0.16
4	*GAK/DGKQ*	rs11248051 [5]	858332	0.11	1.46	3E-9	1.32	0.10	3E-4	1.14	0.15	0.32	1.38	0.11	8E-5
4	*GAK/DGKQ*	rs11248060 [1]	964359	0.12	1.35	2E-9	1.23	0.09	5E-3	1.05	0.13	0.72	1.29	0.10	1E-3
4	*BST1*	rs11724635 [3]	15737101	0.45	0.87	2E-8	0.89	0.04	0.01	0.95	0.07	0.53	0.86	0.04	3E-3
4	*SCARB2*	rs6812193 [4]	77198986	0.37	0.84	8E-10	0.96	0.05	0.42	1.02	0.08	0.79	0.95	0.05	0.36
4	*SNCA*	rs356219 [3]	90637601	0.39	1.30	1E-26	1.37	0.07	1E-10	1.39	0.11	3E-5	1.36	0.07	4E-9
4	*SNCA*	rs356220 [5]	90641340	0.40	1.38	3E-11	1.38	0.07	3E-11	1.40	0.11	2E-5	1.37	0.07	1E-9
4	*SNCA*	rs2736990 [6]	90678541	0.49	1.23	2E-16	1.30	0.06	5E-8	1.37	0.10	5E-5	1.28	0.06	1E-6
4	*SNCA*	rs356198 [1]	90682504	0.18	0.82	5E-9	0.79	0.05	1E-4	0.76	0.08	0.01	0.78	0.05	3E-4
6	*HLA*	rs2395163 [1]	32387809	0.20	0.81	3E-11	0.80	0.05	2E-4	0.87	0.09	0.16	0.78	0.05	9E-5
6	*HLA*	rs3129882 [5]	32409530	0.43	1.26	2E-10	1.31	0.06	3E-8	1.12	0.09	0.15	1.38	0.07	5E-10
7	*GPNMB*	rs156429 [2]	23306020	0.40	0.89	3E-10	0.91	0.04	0.04	0.87	0.07	0.09	0.91	0.05	0.07
8	*FGF20*	rs591323 [2]	16697091	0.27	0.88	7E-11	0.92	0.05	0.13	0.90	0.08	0.22	0.93	0.05	0.19
8	*MMP16*	rs60298754 [2]	89373041	0.02	1.38	2E-9	1.04	0.16	0.78	1.15	0.28	0.58	1.00	0.17	0.98
10	*ITGA8*	rs7077361 [7]	15561543	0.12	0.88	2E-8	0.94	0.07	0.39	1.00	0.11	0.99	0.93	0.07	0.33
12	*LRRK2*	rs1491942 [3]	40620808	0.21	1.19	3E-8	1.22	0.07	6E-4	1.37	0.12	6E-4	1.17	0.07	9E-3
16	*STX1B*	rs4889603 [2]	30982225	0.41	1.12	3E-12	1.01	0.05	0.90	1.07	0.08	0.40	0.99	0.05	0.91
17	*MAPT*	rs2942168 [3]	43714850	0.22	0.76	4E-19	0.76	0.05	5E-6	0.64	0.07	2E-5	0.79	0.05	3E-4
18	*RIT2*	rs12456492 [1]	40673380	0.34	1.19	2E-10	1.09	0.06	0.10	1.22	0.10	0.02	1.06	0.06	0.28

Included in this table are results of prior GWAS that reached genome-wide significance in whites of European decent, and for which data (genotyped or imputed) were available in NGRC GWAS. BP=position in Build 37. All analyses are adjusted for age, sex, PC1, and PC2.

1. Pankratz N, Beecham GW, DeStefano AL, Dawson TM, Doheny KF, Factor SA, Hamza TH, Hung AY, Hyman BT, Ivinson AJ *et al*: **Meta-analysis of Parkinson's disease: identification of a novel locus, RIT2**. *Ann Neurol* 2012, **71**(3):370-384.
2. International Parkinson's Disease Genomics C, Wellcome Trust Case Control C: **A two-stage meta-analysis identifies several new loci for Parkinson's disease**. *PLoS genetics* 2011, **7**(6):e1002142.
3. Nalls MA, Plagnol V, Hernandez DG, Sharma M, Sheerin UM, Saad M, Simon-Sanchez J, Schulte C, Lesage S, Sveinbjornsdottir S *et al*: **Imputation of sequence variants for identification of genetic risks for Parkinson's disease: a meta-analysis of genome-wide association studies**. *Lancet* 2011, **377**(9766):641-649.
4. Do CB, Tung JY, Dorfman E, Kiefer AK, Drabant EM, Francke U, Mountain JL, Goldman SM, Tanner CM, Langston JW *et al*: **Web-based genome-wide association study identifies two novel loci and a substantial genetic component for Parkinson's disease**. *PLoS genetics* 2011, **7**(6):e1002141.
5. Hamza TH, Zabetian CP, Tenesa A, Laederach A, Montimurro J, Yearout D, Kay DM, Doheny KF, Paschall J, Pugh E *et al*: **Common genetic variation in the HLA region is associated with late-onset sporadic Parkinson's disease**. *Nat Genet* 2010, **42**(9):781-785.
6. Simon-Sanchez J, Schulte C, Bras JM, Sharma M, Gibbs JR, Berg D, Paisan-Ruiz C, Lichtner P, Scholz SW, Hernandez DG *et al*: **Genome-wide association study reveals genetic risk underlying Parkinson's disease**. *Nat Genet* 2009, **41**(12):1308-1312.
7. Lill CM, Roehr JT, McQueen MB, Kavvoura FK, Bagade S, Schjeide BM, Schjeide LM, Meissner E, Zauft U, Allen NC *et al*: **Comprehensive Research Synopsis and Systematic Meta-Analyses in Parkinson's Disease Genetics: The PDGene Database**. *PLoS genetics* 2012, **8**(3):e1002548.
